# Dysregulated Ca^2+^ signaling, fluid secretion, and mitochondrial function in a mouse model of early Sjögren’s syndrome

**DOI:** 10.1101/2024.03.19.585719

**Published:** 2024-03-19

**Authors:** Kai-Ting Huang, Larry E. Wagner, Takahiro Takano, Xiao-Xuan Lin, Harini Bagavant, Umesh Deshmukh, David I. Yule

**Affiliations:** 1 Department of Pharmacology and Physiology, University of Rochester, 601 Elmwood Avenue, Rochester, NY. 14526; 2 Arthritis and Clinical Immunology, Oklahoma Medical Research Foundation, 825 NE 13^th^ Street, Oklahoma City, OK 73104

**Keywords:** Salivary gland hypo-function, Xerostomia, Sjögren’s syndrome, DMXAA-induced SS mouse model, Ca^2+^ signaling, TMEM16a channel, mitochondrial dysfunction

## Abstract

Saliva is essential for oral health. The molecular mechanisms leading to physiological fluid secretion are established, but factors that underlie secretory hypofunction, specifically related to the autoimmune disease Sjögren’s syndrome (SS) are not fully understood. SS-like disease was induced by the treatment with 5,6-Dimethyl-9-oxo-9H-xanthene-4-acetic acid (DMXAA), an activator of the stimulator of the interferon gene (STING) pathway. This mouse model mimics exposure to foreign cytoplasmic ribonucleotides occurring following viral and bacterial infection and thought to be an initiating event in SS. Neurotransmitter-stimulated increases in cytoplasmic [Ca^2+^] are central to stimulating fluid secretion, primarily by increasing the activity of the Ca^2+^-activated Cl^-^ channel, TMEM16a. Paradoxically, in DMXAA-treated mice *in vivo* imaging demonstrated that neural-stimulation resulted in greatly enhanced Ca^2+^ levels when a significant reduction in fluid secretion was observed. Notably, in the disease model, the spatiotemporal characteristics of the Ca^2+^ signals were altered to result in global rather than largely apically confined Ca^2+^ rises observed physiologically. Notwithstanding the augmented Ca^2+^ signals, muscarinic stimulation resulted in reduced activation of TMEM16a, although there were no changes in channel abundance or absolute sensitivity to Ca^2+^. However, super-resolution microscopy revealed a disruption in the localization of Inositol 1,4,5-trisphosphate receptor Ca^2+^ release channels in relation to TMEM16a. Appropriate Ca^2+^ signaling is also pivotal for mitochondrial morphology and bioenergetics and secretion is an energetically expensive process. Disrupted mitochondrial morphology, a depolarized mitochondrial membrane potential, and reduced oxygen consumption rate were observed in DMXAA-treated animals compared to control animals. We report that early in SS disease, dysregulated Ca^2+^ signals lead to decreased fluid secretion and disrupted mitochondrial function contributing to salivary gland hypofunction and likely the progression of SS disease.

## Introduction

Saliva plays crucial roles in oral health, including lubricating the mouth, maintaining pH balance, defense against microorganisms, aiding taste, and initiating digestion of macronutrients (Carpenter, 2013; Pedersen et al., 2018; Williamson, 2001). Saliva is produced primarily by three major salivary glands; the submandibular gland (SMG), parotid gland (PG), sublingual gland (SLG), and some minor glands in the lower lip, tongue, and cheeks (Kessler & Bhatt, 2018). Saliva is generated in secretory acinar cells, with its content adjusted by ducts before reaching the mouth. The acinar cells are fundamental to the production of the primary salivary secretion (de Paula et al., 2017). The fluid secretion process is driven by the trans-epithelial movement of Cl^-^ across acinar cells. To accomplish vectorial movement of Cl^-^, acinar cells are polarized such that the basolateral plasma membrane (PM) faces the interstitium and is adjacent to blood vessels, while the apical PM forms a lumen with the distinct PM regions physically segregated by tight-junctional complexes. At the basolateral PM, Cl^-^ are transported into the acinar cell cytoplasm against their electrochemical gradient *via* the Na^+^/K^+^/2Cl^-^ cotransporter, (NKCC1). Following mastication or the experience of the taste and the smell of food, the neurotransmitter, acetylcholine (ACh) is released from parasympathetic nerves and acts on muscarinic receptors on the basolateral PM. Activated muscarinic receptors promote the production of inositol 1,4,5 trisphosphate (IP_3_), and subsequently Ca^2+^ release from endoplasmic reticulum (ER) stores *via* IP_3_ receptors (IP_3_Rs) situated in the ER in the extreme luminal aspects of the cell. Elevated [Ca^2+^]_i_ activates a Ca^2+^-activated Cl^-^ channel named TMEM16a that allows Cl^-^ to move through the apical PM to the ductal lumen which is continuous with the salivary intercalated duct (Melvin et al., 2005; Pedersen et al., 2018). In turn, Na^+^ moves through the paracellular space to balance the Cl^-^ and water follows osmotically both paracellularly and through the water channel aquaporin5 (AQP5) to generate the primary saliva (Soren Nielsen & Birgitte Monster Christensen, 1997; V. Gresz, 2001).

The importance of saliva is underappreciated in the absence of hypofunction. Reduced salivary secretion is termed xerostomia and can result from the iatrogenic effects of drugs, as collateral damage to salivary glands following radiotherapy for malignancy in the head and neck area, and commonly in Sjögren’s syndrome (SS) (Saleh et al., 2015). SS is a chronic autoimmune disorder, that is predominantly manifested as profound dry eye and dry mouth as ultimately the immune system targets and destroys lacrimal and salivary gland cells (Brito-Zeron et al., 2016; Clio P. Mavragani MD, 2014; Khalid F. Tabbara, 2000; Ramos-Casals et al., 2012). SS can occur independently (primary SS, pSS) or concurrently with diseases such as arthritis or lupus (secondary SS, sSS) (Felten, Meyer, & Gottenberg, 2023; Negrini et al., 2022; Sebastian, Szachowicz, & Wiland, 2019; Weerasinghe & Jayasinghe, 2022). SS affects millions of people, predominantly women in their fourth and fifth decades of life (Paul Kruszka, 2009). While treatments can alleviate symptoms, there is no cure or intervention to halt its progression. The etiology of SS remains largely unresolved, but it’s believed to result from a combination of genetic, environmental, hormonal, and possibly viral factors, causing an aberrant immune response directed against the exocrine glands. The identification of SS usually is scored by the extent of salivary hypofunction, the degree of immune infiltration, evidence of damage to minor salivary glands observed following biopsy, and the presence of autoantibodies, such as anti-SSA (Ro) and Anti-SSB (La) and anti-nuclear antibody (ANA) which are classically found in SS (Jonsson et al., 2018). Notably, however, in the early phases of SS, there is minor immune infiltration and little overt damage to exocrine tissue despite profound hypofunction. Provocatively, these data indicate that loss of secretory tissue *per se* is not the causative event resulting in dryness early in the disease, and further indicates that a defect in the stimulus-secretion coupling mechanism precedes glandular destruction and possibly contributes to the progression of disease.

Over the years, numerous mouse models both genetic and “induced” have been developed to study the pathogenesis of SS, with each exhibiting specific aspects of the human condition, including glandular dysfunction, autoantibody production, and lymphocytic infiltration (Gao et al., 2020; Lee et al., 2012). To investigate the early events in SS, in this study, we concentrated on an SS model induced by activation of the stimulator of the interferon gene (STING) pathway. This is thought to mirror the molecular response to bacterial/viral infection. STING is primarily located in the endoplasmic reticulum (ER) and plays a crucial role in the innate immune response, especially against DNA viruses and intracellular bacteria. Activation of STING occurs upon sensing cytosolic DNA as a result of cell damage or from microbial origin following infection. When cytosolic DNA is detected, it is first recognized by a sensor molecule called cGAS (cyclic GMP-AMP synthase). Binding to DNA prompts cGAS to generate cGAMP (cyclic GMP-AMP), which, in turn, binds to and activates STING (Decout et al., 2021). Once STING is activated, it undergoes a series of transformations that ultimately result in the transcription of type I interferon genes, especially interferon-β (IFN-β) (J. Papinska, 2018). The production of type I interferons, especially IFN-β, is a primary antiviral response, and a significant characteristic of SS (Huijser et al., 2022; J. Papinska, 2018). STING can be activated pharmacologically by exposure to 5,6-Dimethyl-9-oxo-9*H*-xanthene-4-acetic acid (DMXAA), which faithfully reproduces the immune response observed following STING activation (Ceron et al., 2019; Gao et al., 2013; Weiss et al., 2017).

In this study, we investigated the early events in the initiation of SS-like disease that lead to salivary gland hypo-function using the DMXAA SS model. We first utilized *in vivo* intravital imaging to investigate any potential dysregulation of Ca^2+^ signaling in the DMXAA-induced SS mouse model. Paradoxically, the Ca^2+^ levels achieved following neural stimulation in mice treated with DMXAA were enhanced despite significantly reduced fluid secretion. Notably, however, the stereotypical spatial characteristics of the Ca^2+^ signal were disrupted. Downstream of the Ca^2+^ signal, the activity of the TMEM16a Ca^2+^-activated Cl channel stimulated by muscarinic secretagogues was reduced, despite no changes in the abundance or localization of the protein or absolute sensitivity to activation by Ca^2+^. The intimate localization of IP_3_R and TMEM16a was however disrupted, which may contribute to the reduced activity of TMEM16a upon agonist stimulation. Moreover, we observed disrupted mitochondrial morphology, abundance, and function in the disease model. These data suggest that early in SS, reduced fluid secretion occurs because of a defect in the secretagogue activation of Cl^-^ secretion. Further significant mitochondrial dysfunction is evident, likely as a result of the aberrant Ca^2+^ signals which may contribute to the progression of SS disease.

## Results

### Saliva secretion is attenuated in both SMG and PG in the SS mouse model

**Figure 1.**
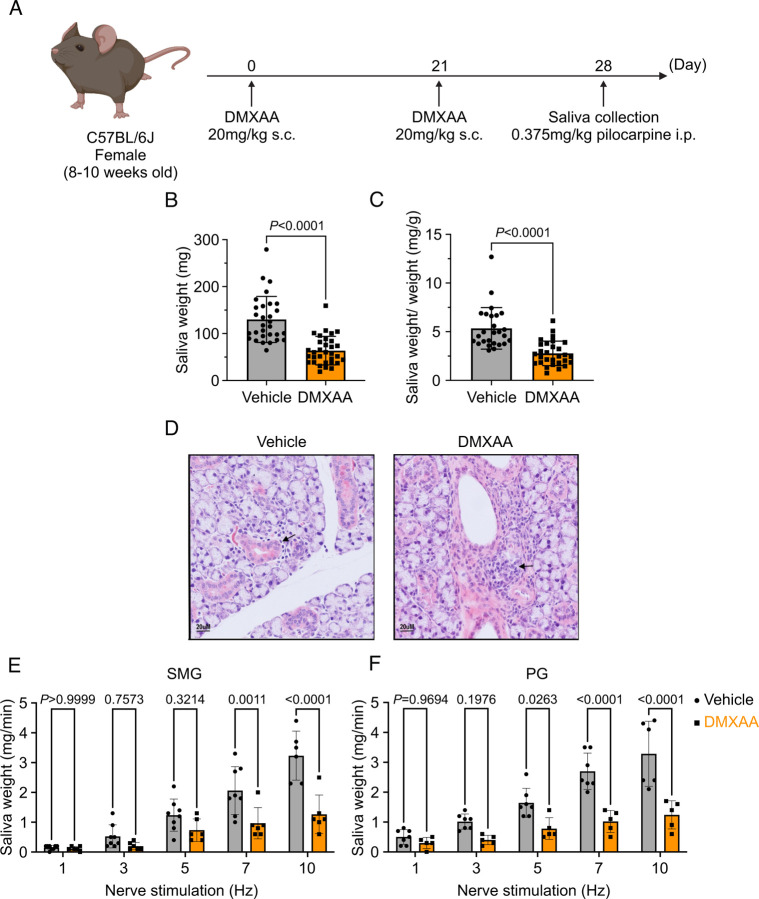
Deficiency in salivary secretion in DMXAA-induced SS mouse model. (A) Schematic timeline for the generation of the SS mouse model. Female wild-type (WT) mice were administered two subcutaneous doses of DMXAA on Day 0 and Day 21. Salivary gland function was assessed on day 28. (B-C) Saliva, stimulated by pilocarpine, was collected over 15 minutes. (B) The amount of saliva secretion was determined by measuring the saliva weight. Vehicle: N= 30 mice, SS mouse model: N= 32 mice. Mean ± SD. (C) The weight of collected saliva was normalized to each mouse’s body weight. Vehicle: N= 26 mice, SS mouse model: N= 29 mice. Mean ± SD. Unpaired two-tailed t-test. (D) H&E stained sections from vehicle or DMXAA-treated animals. Treated animals showed minor lymphocyte infiltration and inflammation as focal peri-vascular/peri-ductal lymphocytic sialoadenitis adjacent to normal-looking acini. (E-F) A comparison of total saliva secretion following 1 min stimulations at the indicated frequency (D) from the SMG of mice (Vehicle: N= 8 mice, SS mouse model: N= 6) and (E) from the PG (Vehicle: N= 7 mice, SS mouse model: N= 5 mice). Mean ± SD. Two-way ANOVA with multiple comparisons.

Activation of the STING pathway in mice has been established as a model for the initiation of SS. This pathway is normally activated following exposure to foreign nucleic acids and is thought to mimic exposure of cells to DNA/RNA from viruses and bacteria. Activation of this pathway in salivary glands is characterized by initiation of a type-1 interferon response, mild immune cell infiltration, and a marked loss of saliva secretion without obvious morphological damage and therefore mimics the early clinical manifestations of SS disease. Thus, to investigate the early cellular events in acinar cells during the initiation of SS in mice, we chose to pharmacologically activate this pathway using DMXAA, a STING pathway agonist. As described in Methods, DMXAA (or control solution) was administered on day 0 and day 21 of the experimental timeline (Figure 1A). Immunofluorescent staining in sliced SMG tissue indicated that STING protein was highly expressed in SMG in the DMXAA-treated mouse on day 28, seven days after the final DMXAA administration (Supplement 1) confirming the activation of the STING pathway. Whole saliva production as a function of stimulation of the major salivary glands was evaluated on day 28 following stimulation by the muscarinic receptor agonist, pilocarpine. To avoid potential weight-related variations in saliva secretion, the total saliva output was normalized to the individual mouse’s body weight. Notably, the average saliva production was reduced from 130.1± 48.96 mg in vehicle-treated mice to 63.71± 30.41 mg in DMXAA-treated mice, a reduction in saliva production of 48.97% (Figure 1B). Consequently, DMXAA treatment resulted in 51.99% saliva production compared to vehicle-treated mice (Figure 1C). Moreover, the H&E staining indicated that mild immune infiltration was observed in the DMXAA-treated mice (Figure 1D). Collectively, these results suggest that DMXAA-treated mice exhibit characteristics of early-stage SS and could be a useful model for investigating the pathophysiological mechanisms underlying secretory dysfunction and advancing our understanding of the disease’s progression.

To further investigate the individual relative contribution of the SMG and PG to the decrease in total saliva secretion using a more physiological stimulation paradigm, we performed experiments where the nerve bundle innervating a particular gland was electrically stimulated and saliva secretion quantitated. Previous research in our lab has established the range and parameters for physiological stimulation of secretion (Takano et al., 2021). The production of saliva was significantly diminished at stimulation frequencies of 7 and 10 Hz in SMG (Figure 1E) and at 5, 7, and 10 Hz in the PG (Figure 1F) in DMXAA-treated animals compared with vehicle controls. These findings confirm that activation of the STING pathway reduces the function of both SMG and PG, consistent with the reduction in the production of whole saliva. Notably, the reduction in function of PG, the gland responsible for the majority of stimulated saliva secretion, was relatively greater than in SMG.

### Altered spatiotemporal characteristics of Ca^2+^ signals in the SS mouse model

**Figure 2.**
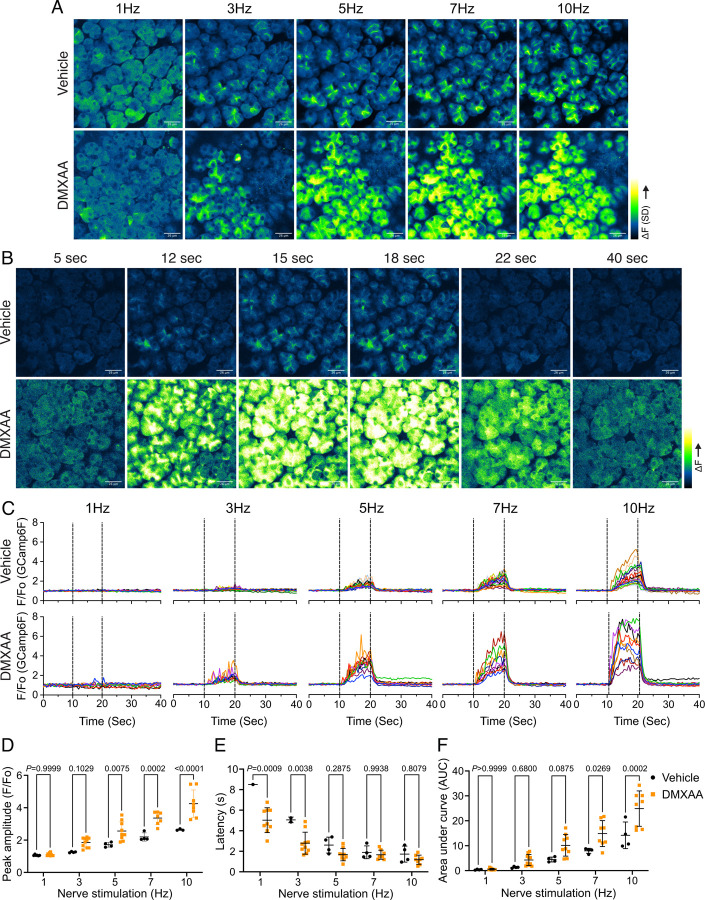
Increased *in vivo* Ca_2+_ signals in SS mouse model. (A) Representative standard deviation images of Ca^2+^ signals during the 10 s of stimulation. Scale bar: 26 μm (B) The time-course of pseudo-color images of Ca^2+^ in response to 7Hz stimulation. Scale bar: 26 μm (C) Representative cellular responses to stimulation at the indicated frequencies. N = 10 cells, one animal. (D) A comparison of peak Ca^2+^, (E) area under curve, and (F) latency during each stimulation in SMG. Each symbol represented the average response of ten cells from one view. Vehicle: N= 3–6 from three mice; SS mouse model: N= 8–10 from four mice. Mean ± SD. Two-way ANOVA with multiple comparisons.

An increase in intracellular Ca^2+^ plays a central role in regulating the intricate cellular machinery underlying secretion processes. In particular, as noted, an increase in Ca^2+^ is important for the activation of ion channels localized in particular domains of the polarized acinar cell which play a central role in the fluid secretion mechanism (Takano et al., 2021). Given that the precise spatiotemporal characteristics of the Ca^2+^ signal in salivary acinar cells are thought to be fundamental to the appropriate activation of the fluid secretion machinery, we evaluated whether the disruption of fluid secretion following DMXAA treatment resulted from dysregulation of the stimulated Ca^2+^ signal. Previous research in our lab developed a platform to study Ca^2+^ signaling *in vivo* using Multiphoton (MP) imaging in transgenic mice engineered to express a genetic-encoded Ca^2+^ indicator, GCaMP6f, specifically in acinar cells (Takano et al., 2021). The protocol for STING pathway induction was applied to the Mist1^CreERT+/−^ GCamp6F^+/−^ genetic mouse (Supplement 2A). The salivary gland function was assessed by the amount of pilocarpine-induced saliva. The secretion deficiency observed in wild-type mice was recapitulated in these mice with a different genetic background (Supplement 2B). We reasoned that decreased fluid secretion in DMXAA-treated mice could result from reduced or dysregulated Ca^2+^ signaling. Therefore, next, we compared the Ca^2+^ signal evoked by direct nerve stimulation in DMXAA-treated versus vehicle control animals *in vivo* in SMG. SMG were stimulated at frequencies optimum for fluid secretion (1–10 Hz) for 10 seconds and the Ca^2+^ signals were recorded. Figure 2A is the standard deviation (SD)-projecting images displayed the Ca^2+^ distribution and amplitude change in the Ca^2+^ signaling following stimulation. In vehicle-treated mice, the Ca^2+^ signals were initiated in a limited number of acinar cells. These signals predominantly propagated below the apical PM. As the stimulation frequency increased, Ca^2+^ signals became more pronounced, and more acinar cells responded. Strikingly, the acinar cells in DMXAA-treated mice demonstrated enhanced sensitivity to stimulation. Even at lower frequency stimulations, a larger number of acinar cells responded, and the Ca^2+^ signals in these cells were notably larger when compared to those in the control group. Figure 2B shows a time series of images following 7 Hz stimulation. Surprisingly, nerve stimulation resulted in a significantly enhanced maximal response when compared to vehicle-treated animals at physiological stimulus frequencies (Figure 2A/B). This augmented response was manifested as an elevated maximum peak [Ca^2+^] (Figure 2D), shorter latency (Figure 2E), and larger area under the curve (AUC) during stimulation in DMXAA-treated animals (Figure 2F).

**Figure 3.**
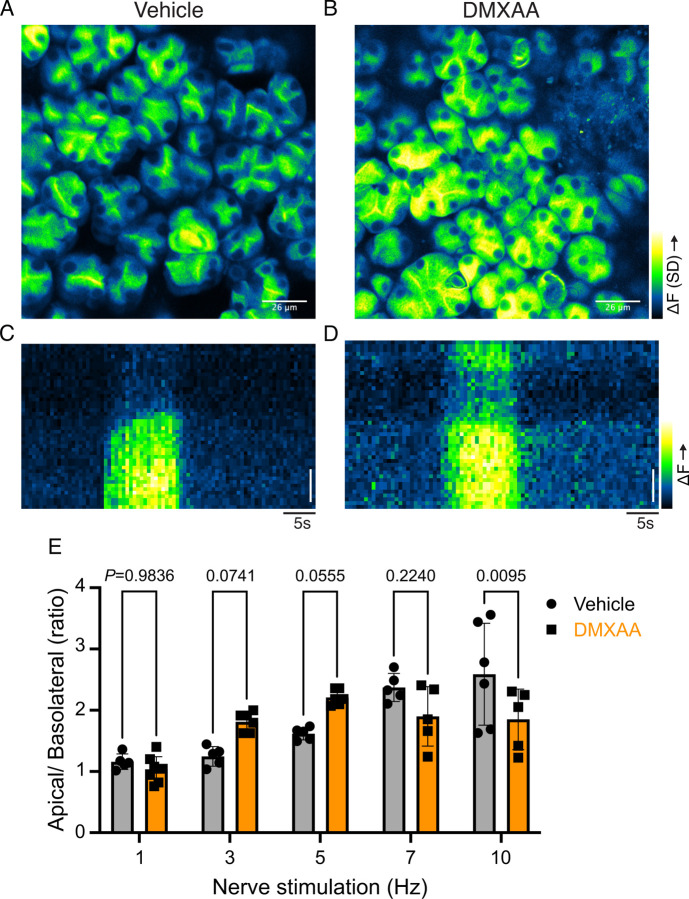
Globalizing *in vivo* Ca_2+_ signals in SS mouse model. (A-B) A representative standard deviation image during the 7Hz stimulation in (A) vehicle condition and (B) in the SS mouse model. Scale bar: 26 μm. (C-D) A representative “kymograph“ image of consecutive lines stacked in space over time for 7Hz stimulation in (C) vehicle condition and (D) SS mouse model. Time is encoded along the X-axis from left to right. Space is encoded along the Y-axis from the apical side (bottom) to the basolateral side (top). Scale bar: 3 μm. (Ε) The ratio of the magnitude of Ca^2+^ signal on the apical side over the basolateral side upon the stimulation at the indicated frequency. Vehicle: N= 5–6 replicates from three mice; SS mouse model: N=5–8 replicates from four mice. Mean ± SD. Two-way ANOVA with multiple comparisons.

In addition to the absolute magnitude of the Ca^2+^ signal, the subcellular spatial characteristics of the stimulated Ca^2+^ rise are also important for appropriate stimulation of fluid secretion (Takano et al., 2021). Physiological Ca^2+^ signals stimulated following nervous stimulation in SMG are invariably initiated in the extreme apical pole of acinar cells and subsequently establish a standing gradient that dissipates rapidly to result in apically confined signals that do not substantially propagate to the basal aspects of the cell following physiological stimulation (Takano et al., 2021). We therefore investigated if the spatial characteristics of stimulated Ca^2+^ signals were altered in DMXAA-treated animals. SD image projections generated during the period of stimulation demonstrated that the [Ca^2+^]_i_ increase was tightly localized below the apical PM within the acinar cells in the vehicle-treated animals (Figure 3A). However, in the DMXAA-treated animals, the [Ca^2+^]_i_ exhibited a more global distribution through the cytoplasm (Figure 3B). The [Ca^2+^]_i_ was visualized *via* line-scan plots revealing the temporal alterations along a line extending from the apical PM to the basolateral PM, traversing the nucleus over time within an acinar cell. A significant [Ca^2+^]_i_ elevation was evident at the basolateral aspects of the acinar cell in the DMXAA-treated animals (Figure 3D) when compared to the vehicle-treated control (Figure 3C). The comparison of Ca^2+^ signal ratios at the apical versus basolateral PM indicated the most significant global Ca^2+^ signal at 10 Hz stimulation (Figure 3E) which corresponds to the stimulation strength that results in maximal fluid secretion (Takano et al., 2021). In summary, these data demonstrate that the absolute magnitude of the Ca^2+^ signal following stimulation is augmented in DMXAA-treated animals and thus cannot in itself account for the reduced fluid secretion. Nevertheless, the spatiotemporal characteristics of the signals are disrupted and could contribute to inappropriate activation of the machinery necessary for ion and fluid secretion.

### Secretagogue stimulated TMEM16a activity is suppressed in the SS mouse model

**Figure 4.**
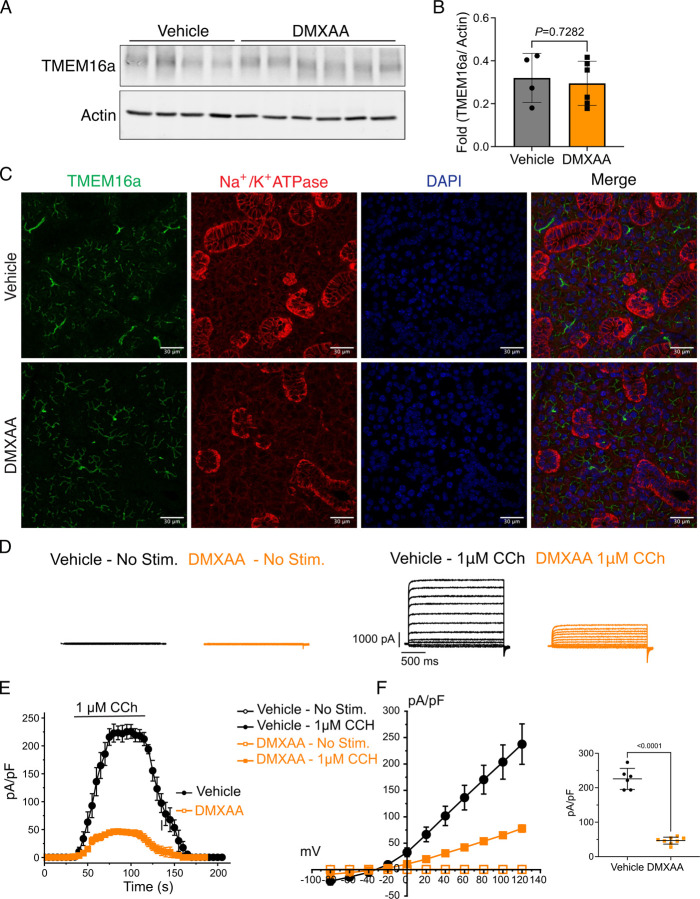
Attenuated whole-cell macroscopic Cl_–_ currents induced by CCh stimulation in SS mouse model. (A) Western blotting showing the protein expression level of TMEM16a in the vehicle condition and the DMXAA-treated SS mouse model. Actin is the internal control. (B) The quantification of TMEM16a protein expression normalized to the internal control, Actin. Vehicle, N= 4 mice; SS mouse model: N= 6 mice. (C) Immunofluorescent staining in SMG tissue for TMEM16a (green), Na^+^/K^+^ ATPase (red), and DAPI for nucleus (blue). The upper panel is from the vehicle-treated control and the bottom panel is from DMXAA-treated animals. Scale bar: 30 μm. Unpaired two-tailed t-test. (D) Cl- currents when cells were held at −50 mV and stepped from −80 to 120 mV in 20 mV increments. (E) Time-dependent Cl^-^ current density changes in response to the CCh in the isolated acinar cells in vehicle conditions and SS mouse model. (F) Current-voltage relationships were measured before and after the addition of CCh in vehicle conditions (N=three mice, 3–4 cells per mouse) and SS mouse model (N=three mice, 3–4 cells per mouse). TMEM16a currents in the treated mice were markedly reduced compared to the control mice. Black dots represent the vehicle-treated cells and orange squares represent DMXAA-treated cells. The open symbols represent no stimulation; the solid symbols represent CCh stimulation.

**Figure 5.**
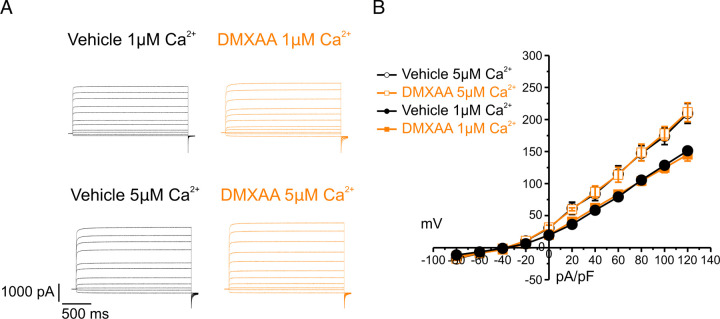
Increased [Ca^2+^]_i_ is capable of restoring TMEM16a functionality to DMXAA-treated mice. Either 1 or 5 μM [Ca^2+^]_i_ in the patch pipette elicited a similar magnitude of Cl^-^ currents for both the treated (N= 3 mice, 3–4 cells per mouse) and control mice (N= 3 mice, 3–4 cells per mouse). (A) Cl- currents when cells were held at −50 mV and stepped from −80 to 120 mV in 20 mV increments. (B) Current-voltage relationships for both populations of mice were essentially identical. Vehicle and SS mouse model: N=3 mice, 3–4 cells per mouse.

**Figure 6.**
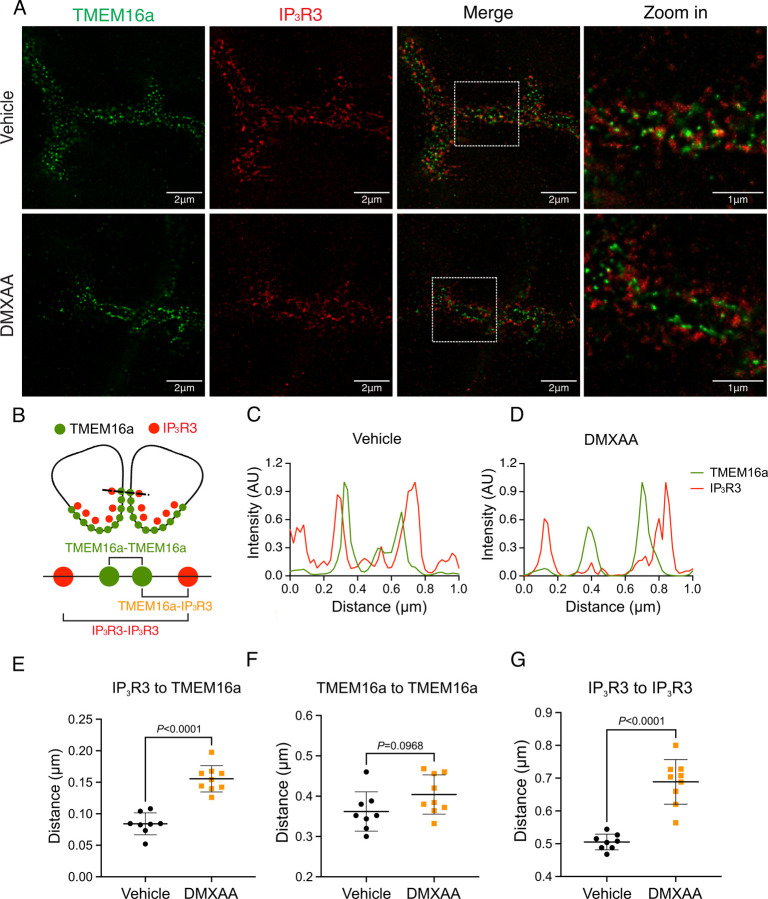
Disrupted proximity between TMEM16a and IP_3_R3 in the DMXAA-treated SS mouse model. (A) Maximum projection of a STED z stack (1 μm) showing TMEM16a (green) and IP_3_R3 (red) in SMG tissue following Huygens deconvolution. The top panel represents the vehicle-treated control, and the bottom panel represents the SS mouse model. Scale bar: 2 μm. Zoomed images highlight the localization of TMEM16a and IP_3_R3 from the white square on the merged images. (B) Diagram illustrating the positioning of apical PM TMEM16a and apical IP_3_R3 in acinar cells. To analyze the proximity, a 1 μm reference line was drawn across the two parallel TMEM16a over two adjacent acinar cells with IP_3_R3 aligned vertically in the cytoplasm. (C-D) The representative traces of changes in fluorescence of TMEM16a (green) and IP_3_R3 (red) over the 1μm distance. (E) Analysis of distance between TMEM16a and IP_3_R3 within cells. (F) Analysis of the distance between parallel TMEM16a on adjacent acinar cells. (G) Distance measurement of apical IP_3_R3 between two cells. Each symbol represents the mean of 5 examinations per image. Vehicle: N= 8 replicates from 3 mice; SS mouse model: N= 9 replicates from 3 mice. Mean ± SD. Unpaired two-tailed t-test.

The rate-limiting step for the secretion of fluid is the activation of the Ca^2+^-activated Cl^-^ channel, TMEM16a. We considered that a reduction in fluid secretion could conceptually occur by a reduction or mislocalization of TMEM16a protein, or by compromised muscarinic receptor-stimulated activation of the channel. Western blotting indicated the TMEM16a protein expression was comparable between the vehicle and SS mouse models (Figure 4A and 4B). In addition, immunolocalization using confocal microscopy demonstrated that TMEM16a localization remained largely unchanged in the SS mouse model (Figure 4C). Thus, a decrease in protein expression or mislocalization of the protein does not result in a reduction in stimulated saliva secretion. We next investigated whether the activation of this ion channel was compromised in DMXAA-treated animals using whole-cell patch clamp electrophysiology. In the absence of stimulation, no Cl^-^ currents were observed in either vehicle or DMXAA animals following either depolarizing or hyperpolarizing voltage steps from a holding potential of −50 mV (Figure 4D). In the presence of 1 μM of muscarinic agonist Carbachol (CCh), robust Cl^-^ currents were measured in acini prepared from vehicle-treated animals (Figure 4E), which were greatly reduced in DMXAA-treated animals (Figure 4D and 4F). Reduced CCh-stimulated Cl^-^ currents could potentially occur because of altered Ca^2+^ regulation of TMEM16a following disruption of the spatial characteristics of the stimulated Ca^2+^ signal. Theoretically, it is also possible that the [Ca^2+^]_i_ in the immediate vicinity of TMEM16a was disrupted, despite the augmented global peak response. We therefore next tested whether TMEM16a activity stimulated directly by either 1 or 5 μM Ca^2+^ in the pipette solution (and thus globally in the cytoplasm) was altered in DMXAA-treated animals. Surprisingly, TMEM16a was activated to a similar extent by Ca^2+^ in the SS mouse model (Figure 5B and 5C). In total, our data suggest that TMEM16a abundance, localization, or activity *per se* are not altered; however, there was a significant reduction in saliva in the DMXAA-treated model. An alternative mechanism would be that the microdomain between the apical ER Ca^2+^ release sites and the apical PM TMEM16a is disrupted in the disease model, resulting in compromised ion channel activation. Therefore, we employed STED super-resolution microscopy to closely examine the spatial relationship between apical PM TMEM16a and IP_3_R3 on the apical ER (Figure 6A). Despite the cell-cell contact distance remaining consistent in the disease model, as indicated by the distance between TMEM16a on the PM of adjacent acinar cells (Figure 6F), a notable increase in distance between the apical TMEM16a and IP_3_R3 expressed on apical ER compared to the control group was observed. In the control mice, the distance between TMEM16a and IP_3_R3 was on average 84 ± 17 nm, versus 155 ± 20 nm in the SS disease mice. Similarly, the distance between IP_3_R3 in adjacent cells was increased from 505 ± 34 nm to 689 ± 68 nm (Figure 6C and 6D). This observation supports the conclusion that the reduced activity of the TMEM16a channel, may be attributable to the disruption of the microdomain between TMEM16a and IP_3_R3, such that the Ca^2+^ flux through the IP_3_R is not communicated appropriately to its effector, TMEM16a.

### Compromised mitochondrial morphology and metabolism in the SS mouse model

**Figure 7.**
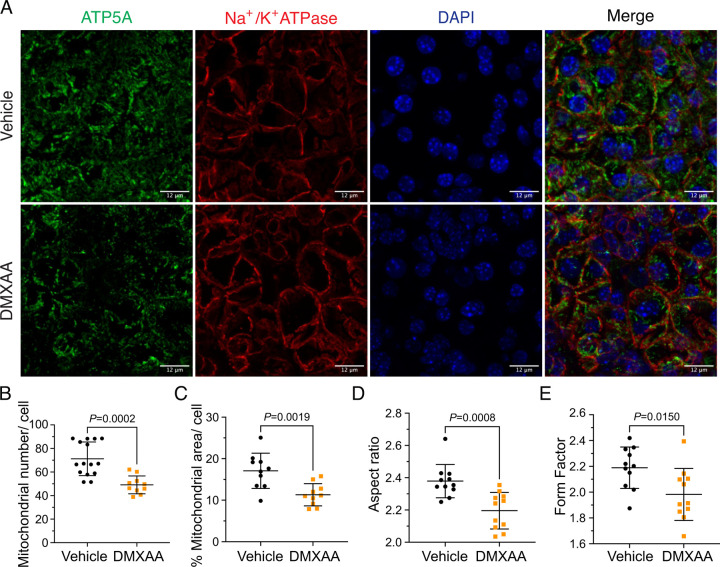
Mitochondrial alterations in acinar cells from the DMXAA-treated SS mouse model. (A) Immunofluorescent staining in SMG tissue for ATP5A (green), Na^+^/K^+^ ATPase (red), and DAPI for nucleus (blue). The upper panel is the vehicle, and the bottom panel is the SS mouse model. Scale bar: 12 μm. The mitochondrial content was quantified by (B) the mitochondrial number per acinar cell and (C) the percentage of area occupied by mitochondria per acinar cell. The mitochondrial morphology was analyzed by the (D) AR for the degree of mitochondrial tubular shape and (E) FF for the degree of mitochondrial branching (complexity). In (B) to (E), black dots represent the vehicle condition, and orange squares indicate the SS mouse model. Each symbol represents the mean of 10 cells per image. Vehicle: N= 10–15 from 3 mice; SS mouse model: N= 10–11 from 3 mice. Mean ± SD. Unpaired two-tailed t-test.

Ca^2+^ modulates cellular metabolism by the intricate bidirectional interaction between the ER and mitochondria. Ca^2+^ transfer between ER and mitochondria is essential for optimal bioenergetics, and dysregulated [Ca^2+^]_i_ can be deleterious to mitochondrial function and alter morphology (Csordas et al., 2006; Duchen, 2000; Katona et al., 2022; Ye et al., 2021). The transfer of Ca^2+^ between ER and mitochondria is dependent on the intimate physical localization of the organelles (Katona et al., 2022). Notably, aberrant mitochondrial morphology has been reported in the salivary glands of SS patients (Barrera et al., 2021). We first investigated mitochondrial abundance and morphology by immunofluorescence staining with antibodies directed against ATP5A, a component of the ATP synthesis machinery to visualize mitochondria, and Na^+^/K^+^ ATPase to localize the plasma membrane (Figure 7A). Using previously published methodologies (Harwig et al., 2018; Valente et al., 2017), quantification revealed a 22.16% ± 4.95 reduction in mitochondrial numbers in the SS mouse model relative to the vehicle-treated control (Figure 7B). Consistent with reduced mitochondrial numbers, less area was occupied by mitochondria in DMXAA-treated acinar cells (Figure 7C). Mitochondria morphology is intricately linked to their bioenergetic status (Duvezin-Caubet et al., 2006; Galloway, Lee, & Yoon, 2012). We next evaluated mitochondrial morphology by their “so-called” aspect ratio (AR) and form factor (FF) in DMXAA and vehicle-treated animals. The AR, the length of the major over minor axes of mitochondria documents the degree of fragmentation or elongation of individual mitochondria. Mitochondria exhibited an 18.35% ± 4.62 decrease in mitochondrial elongation (Figure 7D) and a 20.7% ± 7.78 decrease in mitochondrial branching (Figure 7E) in the disease model compared to the vehicle-treated control condition. Importantly, these changes in mitochondrial number and morphology were not exclusive to the SMG as similar patterns were observed in the PG mitochondria, again marked by reduced mitochondrial count, increased fragmentation, and decreased branching (Supplement 3A-3E).

**Figure 8.**
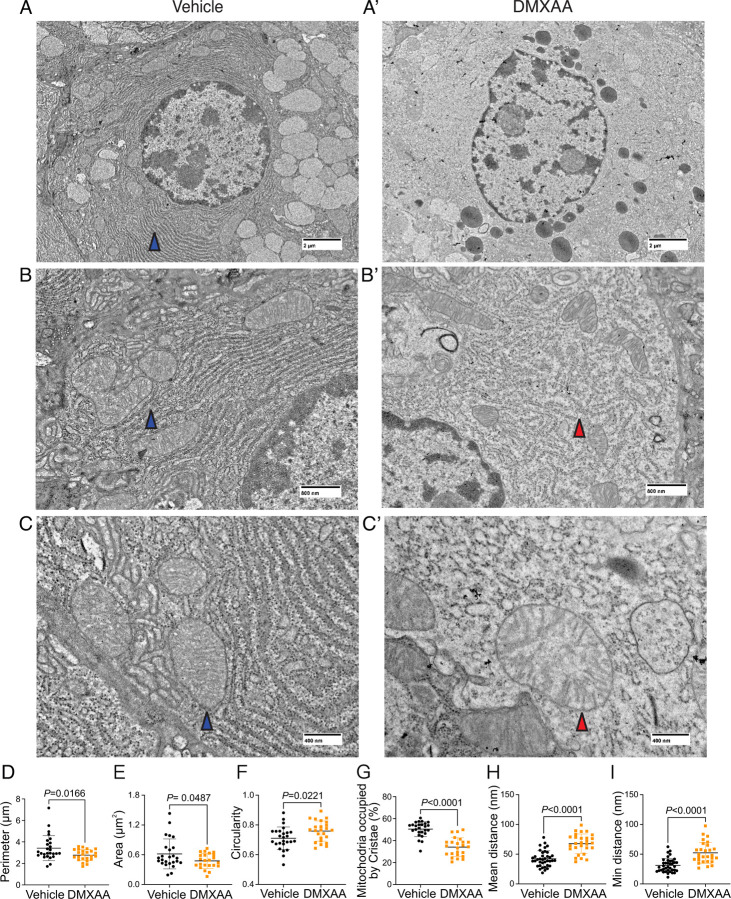
Ultrastructural analysis of mitochondria and ER in SS mouse model. (A-C’’) Images show mitochondrial cristae and ER structure by an EM at scales of (A-A’) 2μm, (B-B’) 800nm, and (C-C’) 400nm. (D) Mitochondrial perimeter, (E) mitochondrial area and (F) circularity were quantified by the shape description in ImageJ. (G) Quantification of mitochondrial cristae dispersion was evaluated by the percentage of cristae occupied in one mitochondrion. The (H) mean and (I) minimum proximity of ER and mitochondria were quantified by the plugin from http://sites.imagej.net/MitoCare/ in ImageJ. Vehicle: N=38 and SS mouse model: N=36 from 3 mice. Mean ± SD. Unpaired two-tailed t-test.

Next, we utilized electron microscopy (EM) to investigate mitochondrial ultrastructure. At low magnification, acinar cells from control mice contained defined mitochondria and well-formed ER stacks (Figure 8A. blue arrow). In contrast, the ER structure was disrupted in the SS disease model (Figure 8A’). At higher magnification, the coordinated ER structure was largely absent in diseased mice (Figure 8B and 8B’), and the close proximity between ER and mitochondria was disrupted (Figure 8H and 8I). Moreover, we also observed scattered mitochondrial cristae at the highest magnification (Figure 8C’ and 8G). Consistent with immunofluorescence studies, quantification of EM micrographs revealed that mitochondria were smaller, more fragmented (Figure 8D and 8E), and rounder (Figure 8F) in shape. In summary, our results collectively indicate significant morphological alterations in mitochondria in the SS disease model.

**Figure 9.**
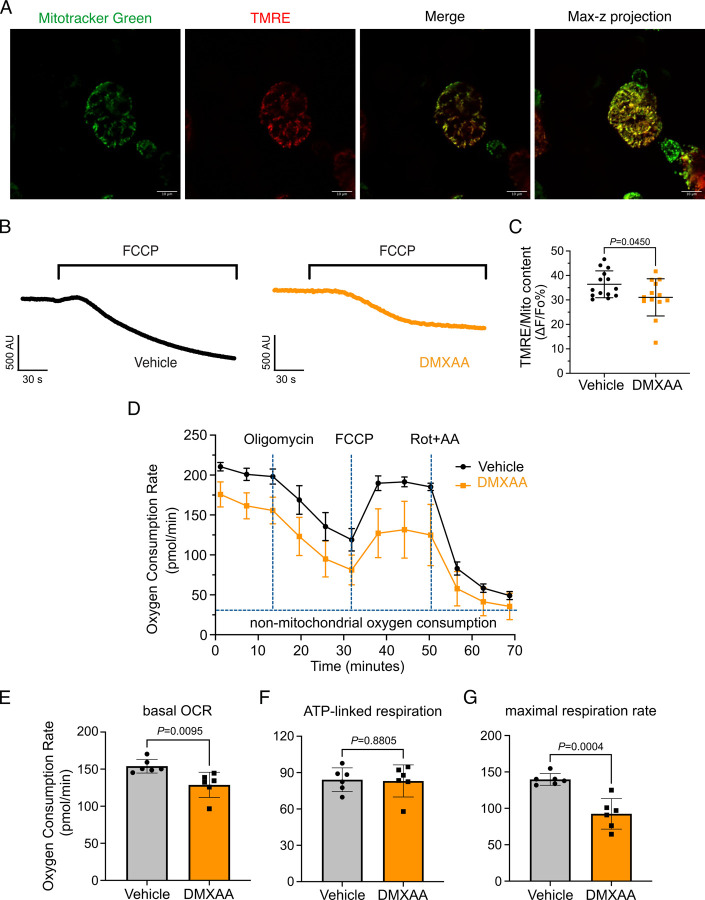
Mitochondrial bioenergetics are compromised in the DMXAA-treated SS model. (A) Mitochondria in the isolated acinar cells were labeled by the MitoTracker Green and co-stained with mitochondrial membrane potential dye, TMRE (red). The merged image shows the colocalization of both dyes, with maximal z-stack projection throughout the acinar cells. (B) Representative changes in mitochondrial membrane potential following FCCP-induced depolarization. The vehicle is shown in black; SS mouse model is in orange. (C) The quantification was achieved by the difference of TMRE normalized to MitoTracker Green. Each dot is the mean of 10 cells from one experiment. Vehicle: N=14 and SS mouse model: N=13 from 3 mice. (D) Real-time mitochondrial respiration function was assessed in isolated acinar cells from the vehicle (black) and SS mouse model (orange) using the Seahorse XFe96 extracellular flux analyzer, in response to the pharmacological mito stress (oligomycin, FCCP, rotenone, and antimycin). Vehicle: N=59 and SS mouse model: N=32 from 6 mice. (E-G) Mitochondrial respiration function parameters were quantified by OCR substracted the non-mitochondrial OCR for (E) basal respiration rate, (F) ATP-linked respiration rate, and (G) maximal respiration rate. Mean ± SD. Unpaired two-tailed t-test.

Mitochondrial morphology is a dynamic process that is intimately associated with mitochondrial bioenergetics and alterations in both occur in response to changes in cellular status (Benard et al., 2007; Galloway, Lee, & Yoon, 2012; Ishihara et al., 2009; Navaratnarajah et al., 2021; Parone et al., 2008). Next, we investigated if changes in morphology might be associated with the disrupted function of mitochondria in the disease model. We, therefore, measured mitochondrial membrane potential (ΔΨ_m_), the driving force of ATP production, in isolated SMG acinar cells. Isolated SMG acinar cells were loaded with TMRE, a ΔΨ_m_-specific dye, and MitoTracker Green, to confirm mitochondrial localization and to facilitate the normalization of indicator loading. The maximal z-stacks projection images taken by confocal microscopy revealed colocalization of TMRE with MitoTracker Green (Figure 9A). To measure the ΔΨ_m_, we quantified the relative maximum dissipation of ΔΨ_m_ in DMXAA and vehicle-treated acinar cells by the mitochondrial uncoupler, FCCP (Figure 9B). TMRE fluorescence normalized against mitochondrial content revealed a marked reduction in ΔΨ_m_ in the acinar cells from the SS disease model (Figure 9C).

An appropriate ΔΨ_m_ mitochondrial membrane potential is vital for maintaining bioenergetics (Zorova et al., 2018). Given that mitochondrial ΔΨ_m_ was significantly depolarized in DMXAA-treated animals, we next evaluated the OCR, a key metric of mitochondrial bioenergetic function in isolated SMG acinar cells. We employed sequential exposure to agents that target the function of the mitochondrial electron transport chain (ETC) using Seahorse technology (Figure 9D). Our results revealed a 25% reduction in basal OCR in the SS model compared to the control animals (at −25.25 ± 7.89 pmol/min; Figure 9E). While ATP-linked respiration showed no significant difference in post-oligomycin-induced ETC Complex V blockade in both conditions (Figure 9F). Intriguingly, the FCCP-provoked maximal respiration rate, an indicator of stress tolerance, remarkably declined by 47% ±9.19 after FCCP treatment in the SS model (Figure 9G). These data indicate impaired mitochondrial function and stress responses in the SS mouse model. In summary, our results indicate that the mitochondrial dynamics and metabolic equilibrium are significantly compromised likely due to disrupted [Ca²⁺]_i_ in the SS disease model.

## Discussion

SS is a complex inflammatory disease resulting from the intersection of genetics and environmental factors. This autoimmune disorder affects exocrine glands including salivary and lacrimal glands, leading to dry mouth and dry eyes, among other symptoms (Brito-Zeron et al., 2016; Clio P. Mavragani MD, 2014; Ramos-Casals et al., 2012). SS animal models are crucial for understanding the pathogenesis, progression, and potential treatments for the disease, though like many animal models of disease, none can recapitulate all the aspects of SS. Currently, SS animal models are categorized as either those derived from genetically modified mice (Kiripolsky et al., 2017; Shen et al., 2009; Shen et al., 2006; Yanfei Hu, 1992) or those where disease is induced by specific agents or environmental factors (Gao et al., 2020; Lee et al., 2012). In the context of SS, DMXAA-induced SS can be used to mimic the early stages of the disease which might be triggered in response to bacterial or viral infection. This model is particularly effective in simulating type-1 interferon immune responses seen in early SS, which is thought to contribute to the initial glandular inflammation (J. Papinska, 2018; Khalid F. Tabbara, 2000; Papinska et al., 2020). The rapid symptom manifestation of disease in the DMXAA-induced model offers an advantage for investigating the early development of SS disease since DMXAA induction is a temporally controlled process, allowing the precise staging of disease onset, thus facilitating studies on the initiating events and ultimately potential early intervention and prevention strategies.

Our studies investigated stimulus-secretion coupling when fluid secretion from SMG and PG in response to physiological stimulation was confirmed to be significantly reduced. Previous work has established a crucial link between an increase in [Ca^2+^]_i_ and stimulation of fluid secretion in the salivary glands (Ambudkar, 2011; Ha-Van Nguyen, 2003; Kiselyov et al., 2006; Lee et al., 1997; Li, Luo, & Muallem, 2004). Efficient secretion is reliant on the specific spatiotemporal regulation of secretagogue-stimulated [Ca^2+^]_i_ signals. Given this idea, our initial hypothesis was that a deficiency in secretion after DMXAA administration could be due to reduced or disrupted secretagogue-stimulated [Ca^2+^]_i_ signals. Indeed, previous work has revealed that in human SS patient acinar cells and the IL14α knock-in transgenic SS mouse model, that CCh-induced [Ca^2+^] signals were diminished. This reduction was attributed to lower expression levels of the IP_3_R2 and IP_3_R3 proteins (Teos et al., 2015). To probe this hypothesis, we employed transgenic Mist1^CreERT2+/–^ x GCaMP6f^+/–^ that expresses Ca^2+^ indicator-GCamp6F specifically in the acinar cells. Firstly, we validated that the activation of the STING pathway leads to similar salivary gland hypofunction in this genetic background (Supplement 2). Surprisingly, however, DMXAA treatment led to a striking increase in the magnitude of neurally-induced [Ca^2+^]_i_ signals. This observation is not consistent with the loss of IP_3_R proteins being responsible for reduced fluid secretion previously reported in other SS models. Indeed, the expression of IP_3_R proteins was unchanged following DMXAA treatment (Supplement 5). The discrepancy could be attributed to the stage of SS disease represented by the early studies, with our data presenting an earlier initiating phase of SS disease prior to progression, at a time point before any notable decrease in IP_3_R proteins has occured. The molecular mechanism responsible for augmented Ca^2+^ signals following DMXAA treatment requires further study, nevertheless, we suggest that this might represent a compensatory mechanism in an effort to drive fluid secretion in the face of compromised physiological stimulus-secretion coupling. Although the Ca^2+^ signals were not reduced, the spatiotemporal characteristics of the Ca^2+^ signal were markedly disrupted. Specifically, during neural stimulation, while in control animals there is a pronounced standing gradient of [Ca^2+^] such that the [Ca^2+^] is much greater in the apical *vs.* basal aspects of the cell, in DMXAA-treated animals this gradient is largely absent as large changes in Ca^2+^ are propagated to the basal regions of the cells. It is conceivable that the alteration in magnitude coupled with changes in the spatial characteristics of the Ca^2+^ signal contributes to both the defect in fluid secretion and downstream cellular changes to ultimately result in the progression of disease.

We investigated whether changes in the secretory machinery *per se* were altered in DMXAA-treated animals to result in hyposecretion. Salivary gland fluid secretion is dependent on TMEM16a facilitating CI^-^ flux across the apical PM as the driving force for water transport paracellularly and through AQP5 (Antonella Caputo, 2008; Soren Nielsen & Birgitte Monster Christensen, 1997; V. Gresz, 2001). The loss of either TMEM16a or AQP5 results in markedly attenuated fluid secretion (Catalan et al., 2015; Romanenko et al., 2010; Tsubota et al., 2001; Zeng et al., 2017). These findings indicate that alteration in expression level, localization, or regulation of these channels could potentially impact fluid secretion. Notably, in DMXAA-treated mice, the AQP5 expression and localization remain unchanged (Supplement 4), consistent with a study in human labial minor salivary glands (Gresz et al., 2015). We next examined if TMEM16a channel function was compromised in the model. Our electrophysiological analysis revealed a significant decrease in TMEM16a activity following CCh-induced stimulation. Again, this reduced activity was not the result of overt mislocalization or lower expression levels of the protein (Figure 4A and 4C). Interestingly, although the secretagogue-stimulated TMEM16a was reduced in acinar cells from DMXAA-treated animals, the sensitivity of the channel to direct activation by Ca^2+^ in the patch pipette appeared to be unaffected. IP_3_R3 Ca^2+^ release channels on the ER are located approximately 50–100 nm from TMEM16a on the PM (Pages et al., 2019). In this microdomain, confocal microscopy cannot easily distinguish the distinct localization of TMEM16a/IP_3_R, despite their localization on different membranes. However, STED super-resolution microscopy provides a much higher spatial resolution, achieving 20–80 nm to enable the differentiation of proteins within 20–80 nm of each other. Data using STED microscopy, suggest that the microdomain between apical ER IP_3_R3 and apical PM TMEM16a is disrupted in the disease model. The severe fragmentation of ER observed in EM images from DMXAA treated animals also is consistent with an alteration in the relationship between ER and other intracellular domains. TMEM16a activation is sensitive to the local Ca^2+^ signal surrounding the channel rather than the global cytoplasmic Ca^2+^ signal (Jin et al., 2016; Shihab Shah, 2020; Wang et al., 2020) and thus it is possible that disruption of this apical microdomain leads to an alteration in the local Ca^2+^ signal that the TMEM16a experiences leading to reduced activation and fluid secretion.

While changes in cytosolic [Ca^2+^] are vitally important for stimulating ion flux and hence fluid secretion, Ca^2+^ is also critical for numerous other physiological processes in salivary gland acinar cells. We focused on the potential effects of the dysregulated Ca^2+^ signal on mitochondrial morphology and function. Secretion is an energy-demanding process, necessitating a constant supply of ATP for numerous functions, including vesicle transport, protein modification, membrane fusion, and maintaining ion gradients. For example, the Na^+^/K^+^ ATPase pump generates the Na^+^ gradient, driving Cl^-^ transport into the cytosol of acinar cells through NKCC1, and SERCA pumps replenish ER Ca^2+^ levels. In this context, mitochondria are essential as they provide ATP, regulate Ca^2+^ homeostasis, supply metabolic intermediates, and coordinate with the ER to orchestrate cellular functions (Jearmine V’tlmart-Seuwen, 1986; Martinez et al., 2020; Messenger, Falkowski, & Groblewski, 2014). Notably, recent studies have highlighted that mitochondria are abundant and display varied positioning and dynamics in salivary gland cells (Porat-Shliom et al., 2019). In SS patients, there are notable alterations in mitochondrial structure, including swelling and disrupted cristae (Barrera et al., 2021; Li et al., 2022). Correspondingly, mitochondrial-related genes, particularly those involved in metabolism, dynamics, and the electron transport complex, are significantly affected (Li et al., 2022). Our data, employing fluorescent immunostaining and EM, mirrors these findings in DMXAA-treated animals. We observed that mitochondrial morphology is altered such that mitochondria are more swollen and rounded, with dispersed cristae, similar to that reported in human SS patients (Barrera et al., 2021). Since optimal mitochondrial bioenergetics are also dependent on Ca^2+^ signals, we assessed mitochondrial function by measuring the mitochondrial membrane potential (ΔΨm) using a membrane potential sensitive probe and the OCR using Seahorse technology. Our results show that in the SS mouse model, ΔΨm, which is critical for ATP synthesis, is diminished. While the ATP-linked OCR remained unchanged, both the basal and maximal OCR were reduced. This suggests that mitochondrial functionality is compromised in the disease model, indicating a decreased capacity to respond to additional cellular stress. An intriguing question arises from these findings: are defects in the function of mitochondria a primary cause of fluid secretion loss in SS, or alternatively is this a consequence of disrupted [Ca^2+^]_i_ regulation? Moreover, DNA from damaged mitochondria can activate the cGAS/STING pathway, leading to inflammation (Kim, Kim, & Chung, 2023; Yu et al., 2020). This implies that compromised mitochondria in early SS stages could trigger prolonged inflammation through the STING pathway, potentially contributing to SS progression. Understanding these mechanisms is crucial for developing effective treatments to halt or slow the progression of SS.

## Material and Methods

All animal procedures were approved by the University of Rochester Committee on Animal Resources (UCAR-2001–214E)

### Animals

The murine model of Sjögren’s syndrome was established through the induction of the STING pathway (Bagavant & Deshmukh, 2020). Briefly, 8–10 weeks old female C57BL/6J wild type (WT) mice (Jackson Laboratory; Jax 000664) received subcutaneous injections of DMXAA (Vadimezan; GC16280) at a concentration of 25 mg/kg of body weight on both day 0 and day 21 of the experimental timeline (see figure 1). The control mouse received vehicle (5% sodium bicarbonate; Sigma-Aldrich; S8761), the DMXAA solvent at the corresponding time points. Experiments were performed on day 28 of the experimental timeline.

### Evaluation of saliva production

The mice were fasted for two hours prior to the evaluation of saliva production. The mice were anesthetized with a solution containing Ketamine (10 mg/mL) and Xylazine (1 mg/ml) by intraperitoneal injection (IP) at a dose of 7 μl/gm body weight over 2 minutes. The mouse was placed on a heating pad at 37℃ during experimentation. A Salimetrics Childen’s swab (Salimetrics; Cat. no. 5001.05) was placed within the oral cavity of each mouse. The mice were administered the muscarinic agonist pilocarpine (0.375 mg/kg body weight; Millipore Sigma; P6503) by IP injection. Two minutes after the pilocarpine injection, saliva was collected for the following 15 minutes. The saliva absorbed was subsequently separated from the moist swab through centrifugation at 10,000 rpm for 1 minute. The measurement of saliva weight served as a quantitative evaluation of the efficacy of whole saliva secretion. To measure neurotransmitter-stimulated saliva secretion more directly, the mouse was anesthetized as previously described (Takano et al., 2021) and a surgical incision was made in the skin to expose the submandibular gland (SMG). The surrounding connective tissue was excised to facilitate positioning within a custom-made 3D-printed gland holder. A pair of stimulation electrodes were attached to the duct bundle and the SMG. The pre-weighed filter paper was positioned within the oral cavity of the mouse to capture saliva secretion. Secretion was initiated by electrical stimulation sequences generated by a stimulus isolator (Iso-flex, A.M.P.I.) set at 5 mA, 200 ms, at frequencies of 1, 3, 5, 7, and 10 Hz with train frequency and duration (typically 1 minute) controlled by a train generator (DG2A, Warner Instruments). The interval between each stimulus was 3 minutes. After stimulation, the filter paper was removed and weighed. The difference between the weight of filter paper before and after the electrode stimulation represented the saliva produced by the respective salivary gland during the given stimulation period.

### *In vivo* Ca^2+^ imaging

Mist1^CreERT2+/–^ x GCaMP6f^+/–^ transgenic mice served as the experimental subjects for Ca^2+^ imaging of the submandibular gland (SMG) *in vivo*. The generation of Mist1^CreERT2+/–^ x GCaMP6f^+/–^ transgenic mice by crossing GCaMP6f^flox^ mice (Jackson Laboratory; Jax 028865) with Mist1^CreERT2^ (Jackson Laboratory; Jax 029228, a gift from Dr. Catherine Ovitt, University of Rochester). A week before the DMXAA or 5% sodium bicarbonate injections, tamoxifen (Sigma-Aldrich; T5648) was given to the mice *via* oral gavage at a dose of 0.25 mg/g of body weight for 3 consecutive days to excise the loxP sites flanking the STOP codon allowing expression of the Ca^2+^ indicator within salivary glands. The mice were anesthetized and gland-exposed, as described previously (Takano et al., 2021; Takano & Yule, 2022; Wahl et al., 2023). The immobilized gland was secured within the holder using a cover glass and maintained in Hank’s salt solution (HBSS). Ca^2+^ imaging was conducted *in vivo* via two-photon microscopy using an Olympus FVMPE-RS system equipped with an Insight X3 pulsed laser (Spectra-Physics) utilizing a heated (OKOLab COL2532) 25x water immersion lens (Olympus XLPlan N 1.05 W MP). GCaMP6F was excited at 950 nm and emission collected between495–540 nm, with images captured at 0.5-second intervals following stimulation for 10 seconds with 3 minutes between stimulation periods. Statistical analyses were performed with two-way ANOVA with multiple comparisons using Prism (GraphPad) as indicated in the figure legends.

### Immunofluorescent staining for sliced tissue

Following verification of decreased saliva secretion in mice, glands were processed for immunocytochemistry. Briefly, the isolated salivary glands were fixed in 4% paraformaldehyde at 4℃ overnight. The fixed gland was processed, embedded in paraffin, and subsequently sliced into 5 μm thick sections. Two temperature-induced antigen retrieval protocols were used either based on HIER buffer (10 mM Tris-base, 1 mM EDTA-dehydrate, pH 9.2) or sodium citrate buffer (10 mM sodium citrate, 0.05% Tween 20, pH 6.0). Gland sections were blocked with the 10% donkey serum in 0.2% PBSA (PBS+ BSA) at room temperature (RT) for 1 hour. Sections were incubated with the primary antibody at 4℃ overnight (TMEM16a (Millipore Sigma; P6593; 1:250), Na^+^/K^+^ ATPase (Abcam; ab2872; 1:250), ATP5A (Abcam; ab14748; 1:500), AQP5 (Abcam; ab239904; 1:500), STING (Cell signaling Technology, Cat. 13647; 1:500)). Following washing, the sections were then incubated with the secondary antibody at RT for 1 hour (Donkey anti-rabbit Alexa 488 (Thermo Fisher Scientific; A-21206; 1:500), Donkey anti-mouse Alexa 594 (ThermoFisher Scientific; A-21203; 1:500)). Nuclei were identified by incubation in DAPI (Thermo Scientific^™^; Cat. 62248; 1:1000) at RT for 5 minutes. Tissue sections were mounted using Immu-Mount solution on a slide and then sealed under a coverslip. Images were acquired by Olympus FV1000MP confocal microscopy employing an Olympus UPlanSApo 60x oil immersion objective. The analysis of images was performed using FIJI software. Statistical analyses were performed with a t-test using Prism (GraphPad) as indicated in the figure legends.

### Patch clamp electrophysiology

Acinar cells were allowed to adhere to Cell-tak-coated glass coverslips for 15 minutes before experimentation. Coverslips were transferred to a chamber containing extracellular bath solution (155 mM tetraethylammonium chloride to block K^+^ channels, 2 mM CaCl_2_, 1 mM MgCl_2_, 10 mM HEPES, pH 7.2). Cl^-^ currents in individual cells were measured in the whole cell patch clamp configuration using pClamp 9 and an Axopatch 200B amplifier (Molecular Devices). Recordings were sampled at 2 kHz and filtered at 1 kHz. Pipette resistances were 3–5 MΩ, and seal resistances were greater than 1 GΩ. Pipette solutions (pH 7.2) contained 60 mM tetraethylammonium chloride, 90 mM tetraethylammonium glutamate, 10 mM HEPES, 1 mM HEDTA (N-(2-hydroxyethyl) ethylenediamine-N, N’, N’-triacetic acid) and 20 μM CaCl_2_ were used to mimic physiological buffering and basal [Ca^2+^]_i_ conditions (~100 nM Ca^2+^). Free [Ca^2+^] was estimated using Maxchelator freeware. Agonists were directly perfused onto individual cells using a multibarrel perfusion pipette. The pipette solution for the increased basal [Ca^2+^]_i_ contained hEDTA and a free [Ca^2+^]_i_ of 5 mM to induce calcium-activated Cl^-^ currents without the addition of any agonists.

### STED microscopy

3D STED microscopy was performed using an Abberior Instruments Expert Line STED microscope equipped with an Olympus UPLSAPO ×100/1.4NA oil immersion objective. Briefly, lobules <1 mm were isolated following injection of saline beneath the capsule with a 29-gauge needle. The connecting tissue was digested in 0.1 mg/ml collagenase containing image buffer at 37℃ for 5 minutes. Then isolated lobules were fixed in 100% methanone at −20℃ for 5 minutes, and subsequently were blocked with 10% BSA in 0.1% PBST (PBS+ 0.1% Tween20) at RT for 1 hour with a gentle shake. TMEM16a was labeled with anti-TMEM16a primary antibody (Millipore Sigma; P6593; 1:300) and STAR RED, goat anti-rabbit IgG secondary antibody (Abberior, Cat#STRED-1001–500UG; 1:1000). IP_3_R3 were labeled with anti-IP_3_R3 primary antibody (BD Transduction Laboratory; Cat. 610313; 1:200) and Alexa Fluor 594 anti-rabbit IgG secondary antibody (Molecular Probes Cat#A-11037; 1:1000). The tissue was mounted on the slides with Prolong^™^ Gold antifade reagent (Invitrogen; Cat. P36930. Sequential confocal and STED images were obtained following excitation of Alexa Fluor 594 and STAR RED by 594 and 640 nm lasers, respectively. Both fluorophores were depleted in three dimensions with a 775 nm pulsed STED laser. Z-stacks were obtained by collecting images at 50 nm intervals using the 3D STED mode. Rescue STED was employed to minimize the light dosage. Blend mode depth projection images were generated and fluorophore volumes and interfaces between these volumes were analyzed using FIJI.

### Seahorse XF cell mito stress assay

Isolated SMGs were finely minced and subsequently resuspended in a solution composed of 0.5% Bovine Serum Albumin (BSA) in Hank’s Balanced Salt Solution (HBSS). To isolate acinar cells, the minced tissue was incubated in 0.5% BSA/HBSS containing 0.2 mg/ml of collagenase type II (Worthington; LS004204) for 30 minutes. Following this incubation, the suspension of cells was centrifuged at 500 rpm for 1 minute and the cellular pellet was then resuspended in 40 μg/ml of Trypsin inhibitor (Millipore; Cat. 65035) to terminate further digestion. The function of mitochondria was assessed in isolated acinar cells by measurement of oxygen consumption rate (OCR) employing a Seahorse XF Cell Mito Stress Test system (Agilent, USA). Briefly, 10 μl of acinar cells were seeded into individual wells of Seahorse cell culture microplates and the OCR was determined utilizing the Seahorse XFe96 extracellular flux analyzer following sequential exposure to 4μg/ml oligomycin (Millipore Sigma; O4876), 4µM carbonyl cyanide-4 (trifluoromethoxy)phenylhydrazone (FCCP; Millipore Sigma; C2920), and 0.5 µM rotenone/antimycin (Millipore Sigma; R8875; A8674) to measure the quantification of basal respiration, ATP-linked respiration, and maximum respiration rate, respectively. Statistical analyses were performed with, t-test using Prism (GraphPad) as indicated in the figure legends.

### Measurement of mitochondrial membrane potential

Isolated SMG acinar cells were loaded with 20 nM Tetramethylrhodamine, Ethyl Ester (TMRE; ThermoFisher Scientific: T669), and 1μM of MitoTracker Green (Invitrogen^™^; M7514). Fluorescence of both TMRE and MitoTracker Green was captured simultaneously using an inverted epifluorescence Nikon microscope with a 40 X oil immersion objective. The TMRE fluorescence was excited at 560 nm and emitted light collected at 574 nm; MitoTracker Green was excited at 488 nm and emitted light collected at 530 nm. Images were obtained every 1 s with an exposure of 20 ms and 4 × 4 binning using a digital camera controlled by TILL Photonics, TILLvision software. The acinar cells were exposed to 4 μM FCCP for 3 minutes by perfusion to rapidly dissipate the membrane potential. Mitochondrial membrane potential was quantified as the change in the ratio of TMRE/MitoTracker Green fluorescence before and after the administration of FCCP. Statistical analyses were performed with a t-test using Prism (GraphPad) as indicated in the figure legends.

### Western blotting

Finely minced salivary glands were homogenized in a lysis buffer supplemented with protease inhibitor cocktail (Complete mini; Roche Diagnostics) for 16–20 strokes. After incubating on ice for 30 minutes, solubilized proteins were separated by centrifugation at 13000 rpm at 4℃ for 30 minutes. 10μg of protein lysate was loaded on 7.5%- 12% SDS- polyacrylamide gels. Subsequently, the proteins were transferred to PVDF membranes at a voltage of 35V at 4°C overnight. The membrane was blocked with 5% non-fat skimmed milk in TBST (50 mM Tris-HCl, pH 7.5 with 0.1% Tween20) at RT for 1 hour and subsequently incubated with primary antibodies overnight at 4°C (Actin (Millipore Sigma; A2228; 1:10000), IP_3_R2 (Antibody Research Corporation; 1:1000), IP_3_R3 (BD Transduction Laboratory; Cat. 610313; 1:1000), TMEM16a (Abcam; ab84115; 1:1000)). After being washed with 0.1% TBST, the membranes were incubated with secondary antibodies at RT for 1 hour (Goat anti-rabbit IgG (H&L) (Invitrogen; SA535571; 1:10000), Goat anti-mouse IgG (H&L) (Invitrogen; SA535521; 1:10000)). Protein band intensity from western blotting was quantified by FIJI. The relative ratio of DMXAA-treated/ vehicle control was calculated in Excel. Lastly, graphical generation and statistics were performed with a t-test using Prism (GraphPad) as indicated in the figure legends.

## Supplementary Material

Supplement 1 **Supplement 1. Up-regulation of STING protein expression in both SMG and PG treated with DMXAA.** Immunofluorescent staining in SMG tissue for STING (green), Na^+^/K^+^ ATPase (red), and DAPI for nucleus (blue). The upper panel is in vehicle-treated condition and the bottom panel is the SS mouse model. Scale bar: 12 μm. (B-C) STING protein expression was quantified by the percentage of a cell occupied by STING protein in (B) SMG and (C) PG. Vehicle, N= 3 mice; SS mouse model: N= 3 mice. Unpaired two-tailed t-test.
**Supplement 2. Deficiency in secretion in Mist1_CreERT+/−_GCamp6F_+/−_ genetic mouse treated with DMXAA.** (A) Schematic timeline for the generation of the SS mouse model in the Mist1^CreERT+/−^ GCamp6F^+/−^ genetic mouse. The female Mist1^CreERT+/−^Gcamp6F^+/−^ mouse received two subcutaneous doses of DMXAA on day 0 and day 21. The salivary gland function was assessed on day 28. (B) The gland function was evaluated by the weight of pilocarpine-induced saliva, normalized to each mouse’s weight. Vehicle = 3 mice, DMXAA= 4 mice. Mean ± SD. Unpaired two-tailed t-test.
**Supplement 3. Mitochondrial alterations in the parotid gland of SS mouse model.** (A) Immunofluorescent staining in PG tissue for ATP5A (green), Na^+^/K^+^ ATPase (red), and DAPI for nucleus (blue). The upper panel is from vehicle-treated animals and the bottom panel is from the SS mouse model. Scale bar: 12 μm. The mitochondrial content was quantified by (B) the mitochondrial number per acinar cell and (C) the percentage of area occupied by mitochondria per acinar cell. The mitochondrial morphology was analyzed by the (D) AR for the degree of mitochondrial tubular shape and (E) FF for the degree of mitochondrial branching (complexity). In (B) to (E), black dots represent the vehicle condition, and orange squares indicate the SS mouse model. Each symbol represents the mean of 10 cells per image. Vehicle: N= 20 and SS mouse model: N= 15–17 from four mice. Mean ± SD. Unpaired two-tailed t-test.
**Supplement 4. AQP5, the water channel, remained the comparable expression and proper localization in SMG in the disease mouse model.** (A) Immunofluorescent staining in SMG tissue for AQP5 (green), Na^+^/K^+^ ATPase (red), and DAPI for nucleus (blue). The upper panel is from vehicle condition and the bottom panel is from the SS mouse model. Scale bar: 12 μm. (B) Western blotting showed the protein level of AQP5 in SMG from vehicle-treated control and disease mouse model. (C) The quantification of AQP5 normalized to internal control, Actin. Vehicle: N= 4; SS mouse model: N= 6. Mean ± SD. Unpaired two-tailed t-test.
**Supplement 5. No significant alteration in IP_3_R protein levels in SMG in the DMXAA-treated mouse model.** (A) Western blotting indicated the IP_3_R2 and IP_3_R3 protein levels from vehicle-treated control and SS mouse model. Actin was probed as the internal control. (B) The quantification of IP_3_R2 and IP_3_R3 normalized to Actin, the internal control. Vehicle: N= 4; SS mouse model: N= 6. Mean ± SD. Unpaired two-tailed t-test.
