# Supplementary figures and images for: Dysregulated Ca2+ signaling, fluid secretion, and mitochondrial function in a mouse model of early Sjögren’s syndrome
Source: bioRxiv. 2024 Mar 19:2024.03.19.585719. Preprint. [Version 1] doi: 10.1101/2024.03.19.585719 (PMC10983907; doi:10.1101/2024.03.19.585719)

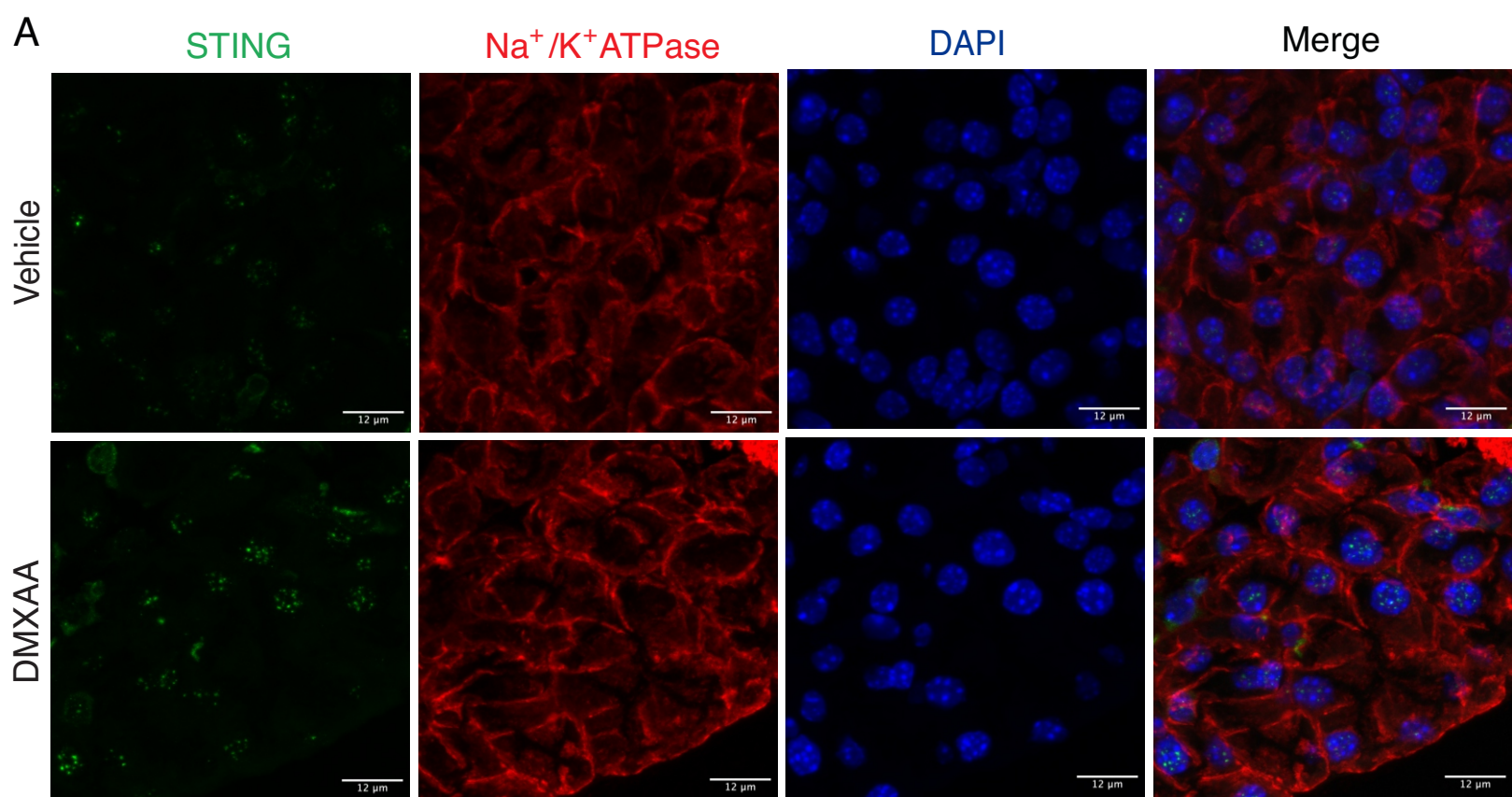

**B**

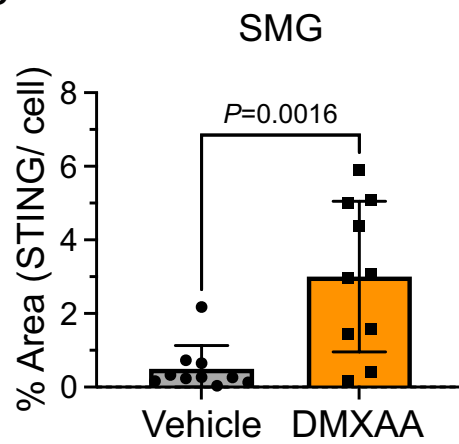

**C**

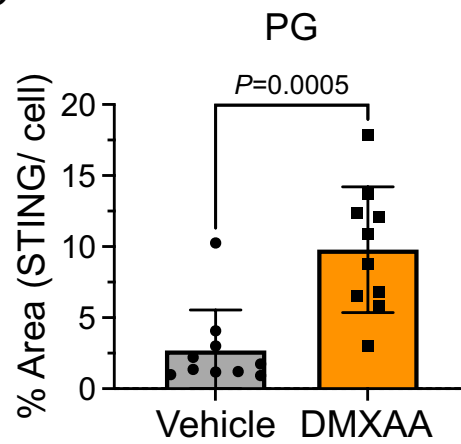

Supp. Fig.1

A

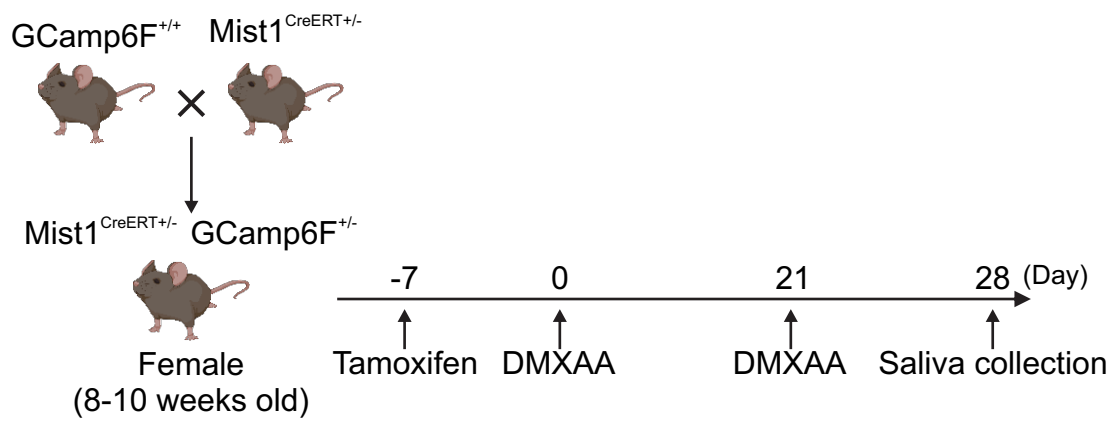

B

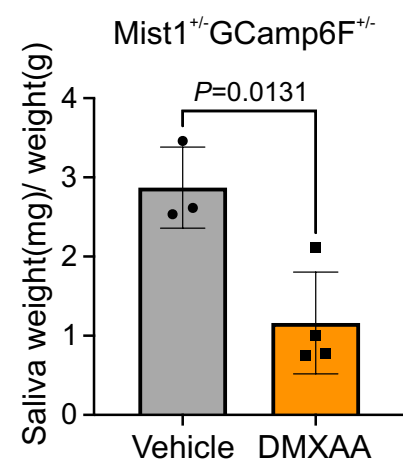

Supp. Fig. 2

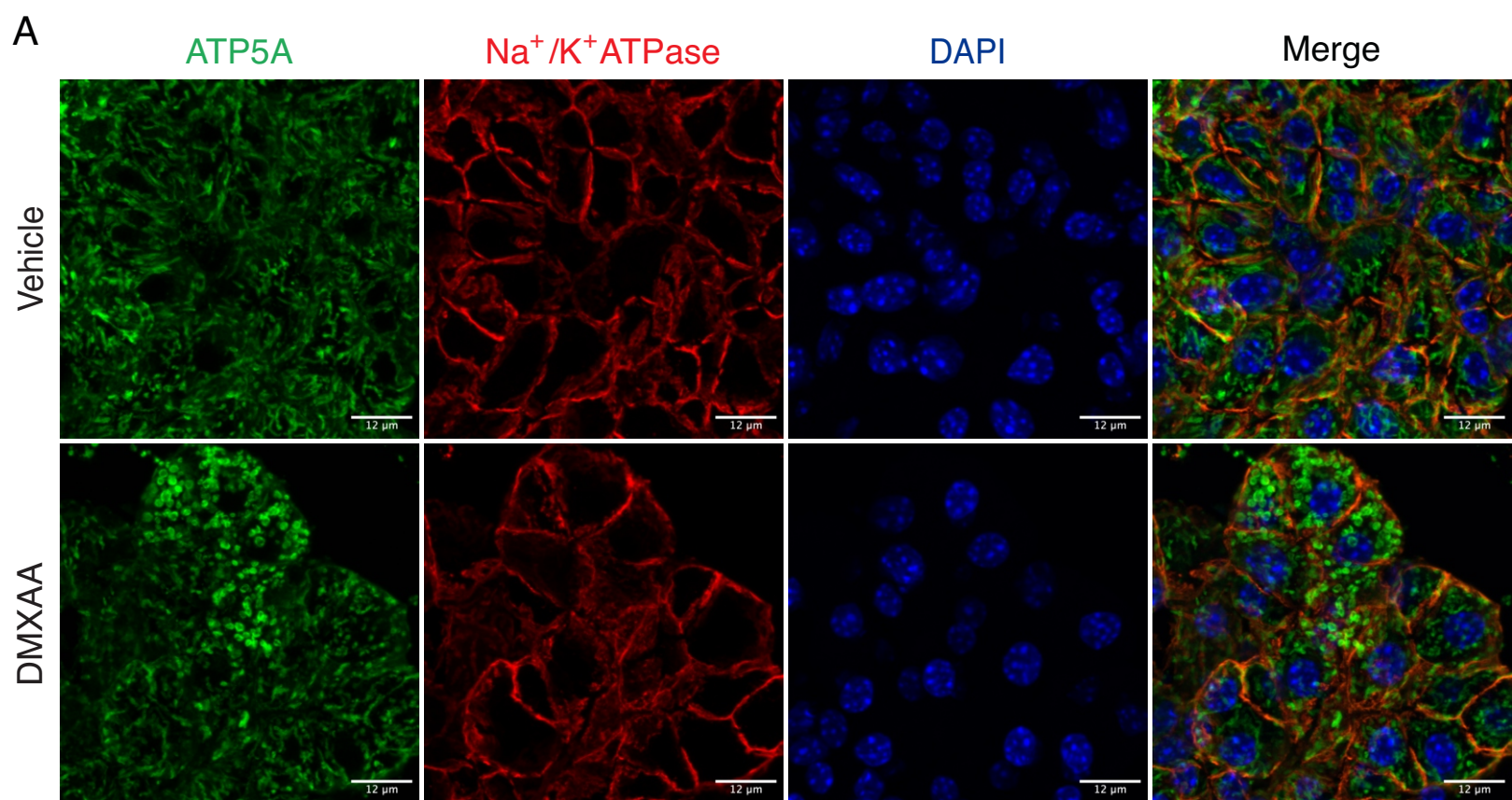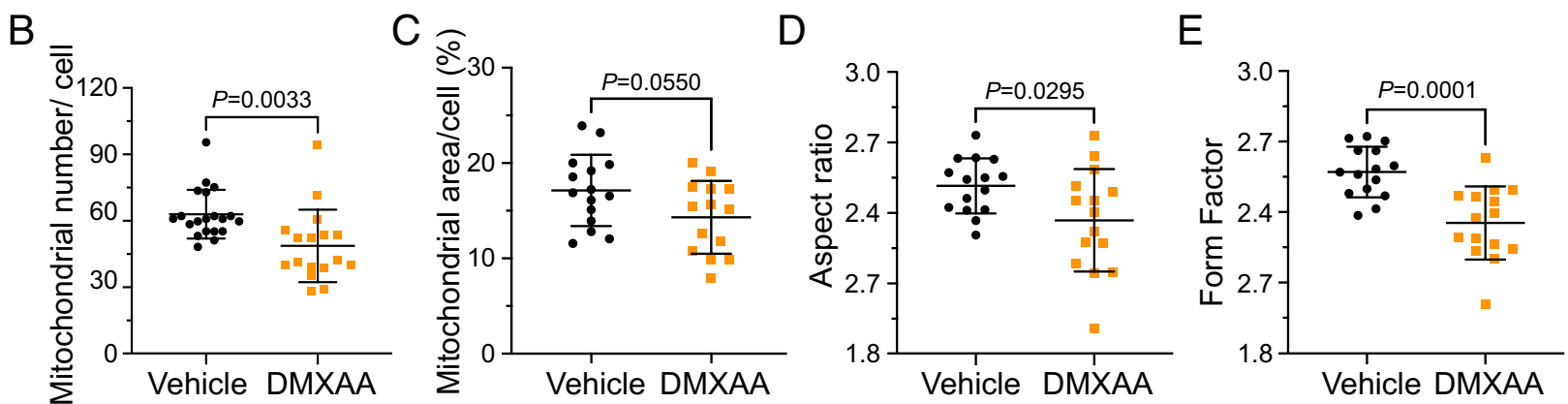

Supp. Fig. 3

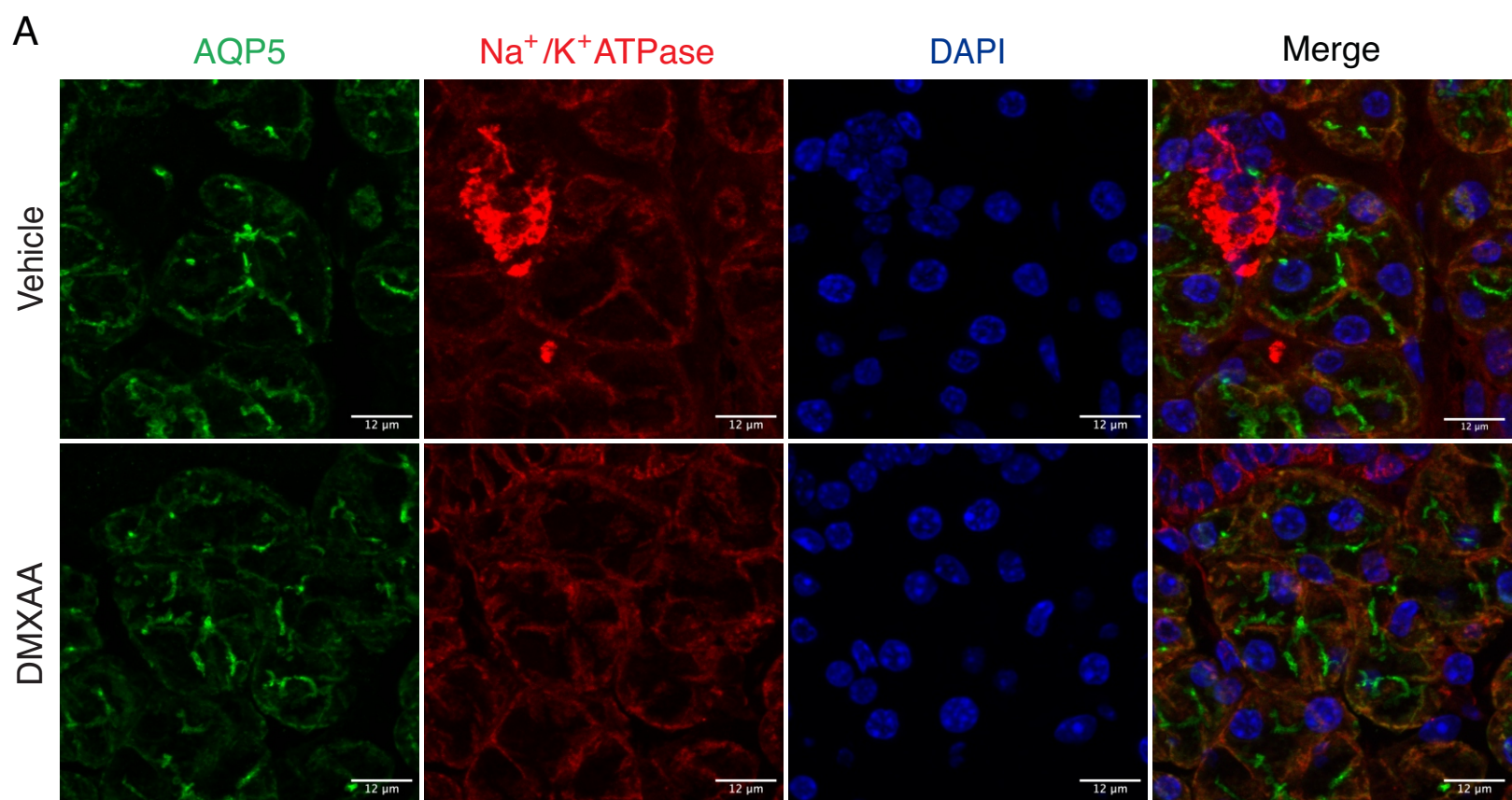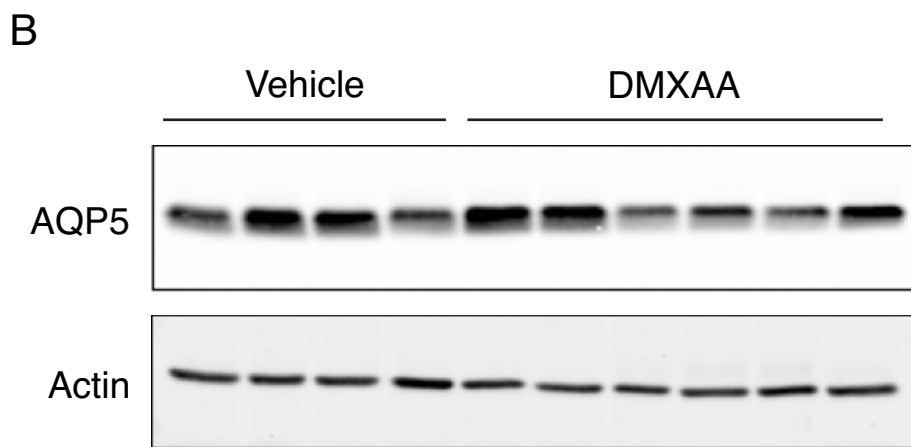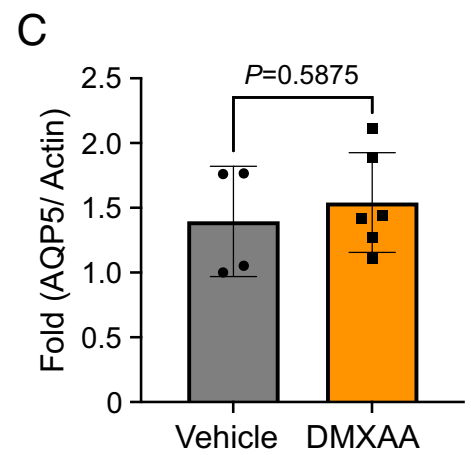

Supp. Fig. 4

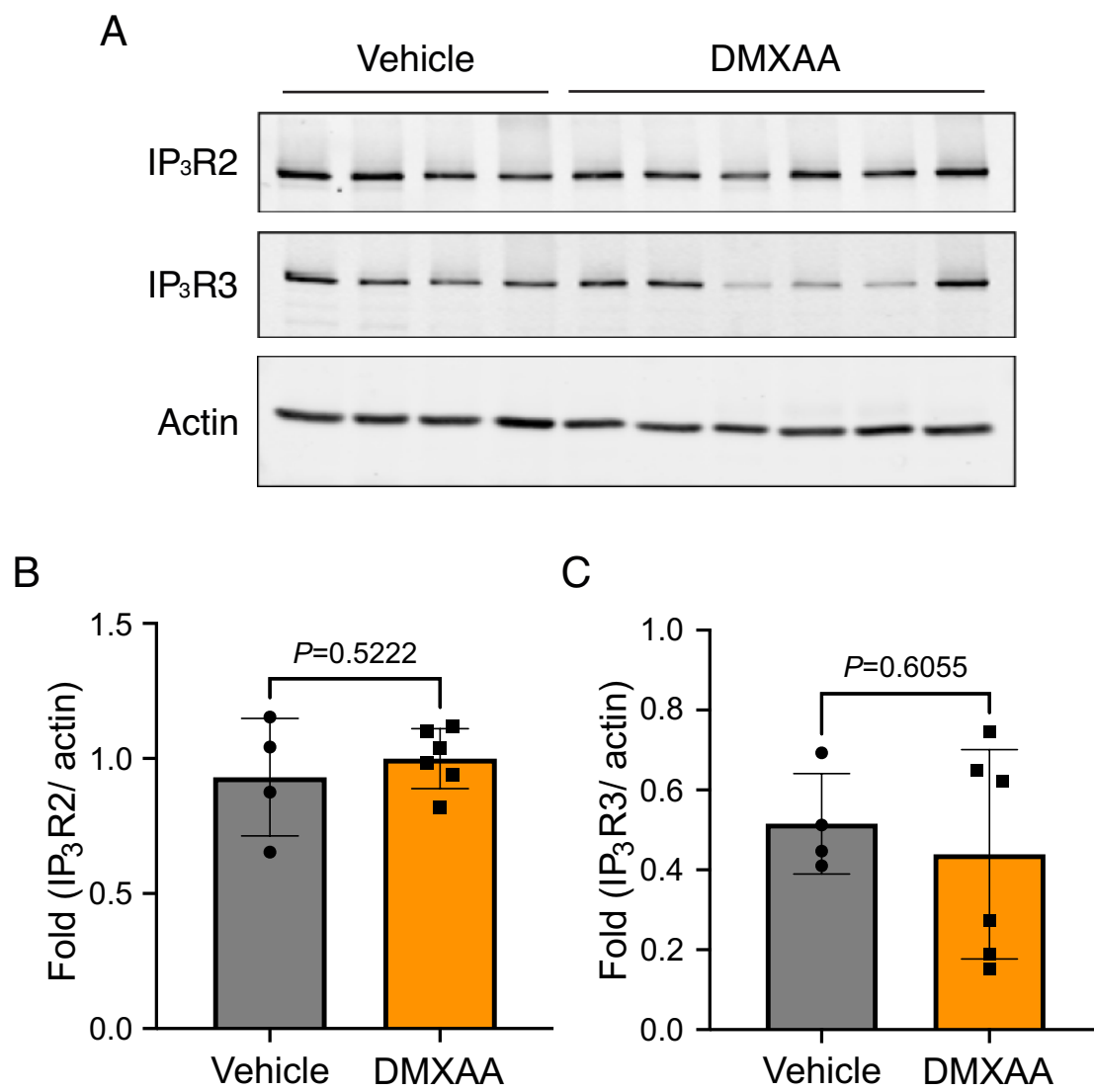

Supp. Fig. 5

Supplement: Supplement 1 — Up-regulation of STING protein expression in both SMG and PG treated with DMXAA. Immunofluorescent staining in SMG tissue for STING (green), Na+/K+ ATPase (red), and DAPI for nucleus (blue). The upper panel is in vehicle-treated condition and the bottom panel is the SS mouse model. Scale bar: 12 μm. (B-C) STING protein expression was quantified by the percentage of a cell occupied by STING protein in (B) SMG and (C) PG. Vehicle, N= 3 mice; SS mouse model: N= 3 mice. Unpaired two-tailed t-test. Supplement 2. Deficiency in secretion in Mist1CreERT+/−GCamp6F+/− genetic mouse treated with DMXAA. (A) Schematic timeline for the generation of the SS mouse model in the Mist1CreERT+/− GCamp6F+/− genetic mouse. The female Mist1CreERT+/−Gcamp6F+/− mouse received two subcutaneous doses of DMXAA on day 0 and day 21. The salivary gland function was assessed on day 28. (B) The gland function was evaluated by the weight of pilocarpine-induced saliva, normalized to each mouse’s weight. Vehicle = 3 mice, DMXAA= 4 mice. Mean ± SD. Unpaired two-tailed t-test. Supplement 3. Mitochondrial alterations in the parotid gland of SS mouse model. (A) Immunofluorescent staining in PG tissue for ATP5A (green), Na+/K+ ATPase (red), and DAPI for nucleus (blue). The upper panel is from vehicle-treated animals and the bottom panel is from the SS mouse model. Scale bar: 12 μm. The mitochondrial content was quantified by (B) the mitochondrial number per acinar cell and (C) the percentage of area occupied by mitochondria per acinar cell. The mitochondrial morphology was analyzed by the (D) AR for the degree of mitochondrial tubular shape and (E) FF for the degree of mitochondrial branching (complexity). In (B) to (E), black dots represent the vehicle condition, and orange squares indicate the SS mouse model. Each symbol represents the mean of 10 cells per image. Vehicle: N= 20 and SS mouse model: N= 15–17 from four mice. Mean ± SD. Unpaired two-tailed t-test. Supplement 4. AQP5, the water [file NIHPP2024.03.19.585719v1-supplement-1.pdf]
